# Identification of plasmalogens in *Bifidobacterium longum*, but not in *Bifidobacterium animalis*

**DOI:** 10.1038/s41598-019-57309-7

**Published:** 2020-01-16

**Authors:** Shiro Mawatari, Yasuhiro Sasuga, Tomomi Morisaki, Mika Okubo, Takako Emura, Takehiko Fujino

**Affiliations:** 1 Institute of Rheological Functions of Food, 2241-1 Kubara, Hisayama-chou, Kasuya-gun, Fukuoka, 811-2501 Japan; 2 Hachioji Center for Research & Development, B&S Corporation Co., Ltd. 1-31-1 Nanakuni, Hachioji-shi, Tokyo 192-0919 Japan

**Keywords:** Bacteriology, Alzheimer's disease

## Abstract

Plasmalogens are glycerophospholipids that contain a vinyl ether bond at the sn-1 position of glycerol backbone instead of an ester bond. Plasmalogens are indicated to have many important functions in mammalian cells. On the other hand, it is suggested that some gut microbiota plays many probiotic functions to human health. Presence of plasmalogens in *Clostridium* strains in gut microbiota is well-known, but presence of plasmalogens in *Bifidobacterium longum* (*B. longum*) strain, one of the most important probiotic gut microbiota, has not been reported. We identified plasmalogens in lipid extract from some *B. longum* species, but not from *Bifidobacterium animalis (B. animalis)* species which are another important strain of probiotic *bifidobacteria*. Major phospholipid classes of plasmalogens in *B. longum* species were cardiolipin, phosphatidylglycerol and phosphatidic acid. Almost all of the phospholipids from *B. longum* examined were indicated to be plasmalogens. Although major phospholipid classes of plasmalogens in human brain and major phospholipid classes of plasmalogens in *B. longum* are different, it is interesting to note that many reported functions of microbiota-gut-brain axis on human neurodegenerative diseases and those functions of plasmalogens on neurodegenerative diseases are overlapped. The presence of plasmalogens in *B. longum* species may play important roles for many probiotic effects of *B. longum* to human health.

## Introduction

Glycerophospholipids consist of diacyl glycerophospholipids and ether glycerophospholipids. Ether glycerophospholipids are characterized by an alkyl or an alkenyl (a vinyl ether-) linkage at the sn-1 position of the glycerol backbone. The glycerophospholipids with alkenyl bond (vinyl ether bond) at the sn-1 position are generally called plasmalogens^1–9^. Plasmalogens are found in almost all mammalian tissues and constitute about 20% of the total phospholipids in the mammalian tissues^1,5^. Predominant ether phospholipids in mammalian tissues are ethanolamine ether phospholipid (ePE) and choline ether phospholipid (ePC)^1–9^^,^ and other ether phospholipids such as serine and inositol ether phospholipid are reported to be less than 0.2% of the total phospholipids^1,5,8^. In mammalian tissues, ethanolamine plasmalogen (plsPE) is 10-fold more abundant than choline plasmalogen (plsPC) except heart and skeletal muscle^1,2,5^. Relative composition of plsPC is high in heart and skeletal muscle. Plasmalogens are abundant in the brain, retina, leukocytes (immune cells), sperm, heart, and skeletal muscle in mammals^1,5^. In these plasmalogens, the aliphatic moieties at the sn-1 position are mainly C16:0, C18:0 or C18:1. On the other hand, the sn-2 position is preferentially occupied by polyunsaturated fatty acids such as arachidonic acid (C20:4) or docosahexaenoic acid (C22:6)^1,2,5^. Plasmalogens are not only a structural component of mammalian cell membrane and a reservoir for second messengers, but also may be involved in membrane fusion, ion transport, cholesterol efflux, and antioxidant for cell membranes^1–8^. Plasmalogens are important for the organization and stability of lipid raft^9,10^.

Plasmalogens are found widespread in vertebrates and invertebrates. However, presence of plasmalogens in fungi and plant cells is not established^11,12^. Plasmalogens are found in some strictly anaerobic bacteria, but not in facultatively anaerobic bacteria or aerobic bacteria^11,12^. It is well known that plasmalogens are found in *Clostridium* strains in gut-microbiota^11–15^. Most of the *Clostridiium* strains are not considered to be probiotic in mammals; however, it is recently reported that some *Clostridium* strains in gut have probiotic effects on human health^16–18^. On the other hand, *Bifidobacterium longum (B. longum)* and *Bifidobacterium animalis (B. animalis)* are considered to be the most important probiotic bacteria in mammalian gut-microbiota^19–21^. It has not been reported that plasmalogens are present in *Bifidobacterium longum* species. We identified plasmalogens in some of *B. longum* species, but not in *B. animalis* species.

## Results

### Differences of phospholipid composition among gut microbiota

**Figure 1:**
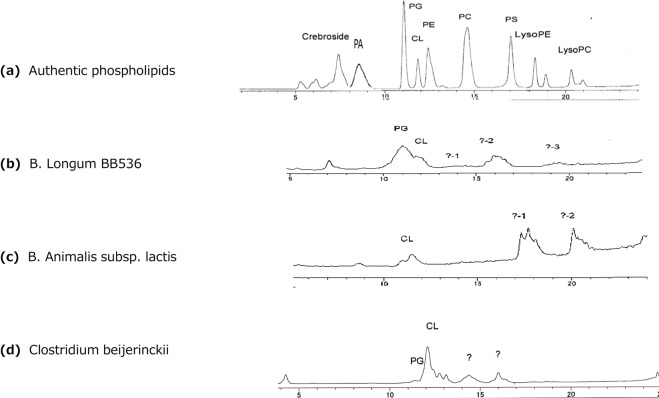
HPLC chromatogram of phospholipids. Authentic phospholipids (**a**), phospholipids of *B. longum* BB536 (**b**), phospholipids of *B. animalis subsp. lactis* (**c**), phospholipids of *Clostridium beijerinckii* (**d**). PA; phosphatidicacid, PG; phosphatidylglycerol, CL; cardiolipin, PE; phosphatdylethanolamine, PC; phosphatidylcholine, PS; phosphatidylserine, LysoPE; lysophosphatidylethanolamine, LysoPC; lysophosphatidylcholine,?; un-identified phospholipid.

The HPLC-ELS D method used in the present study can separate almost all major phospholipids present in animal tissues by a single run of HPLC (Fig. 1a), but phospholipids from bacteria did not separate clearly each other (Fig. 1b–d). Causes of the incomplete separation of bacterial phospholipids by the HPLC-ELSD method were not clear, but may be due to presence of some other unidentified phospholip id classes^22^. However, phospholipid compositions were clearly different among *B. longum, B. animalis* and *Clostridium* (Fig. 1b–d). Furthermore, we experienced that the patterns of phospholipids composition and/or phospholipid classes on the chromatograms of the same species of *B. longum* often changed after re-cultured by the same culture medium after storage at a refrigerator or a deep freezer (−30 °C).

### Presence of plasmalogens in some of *B. longum* species

**Figure 2:**
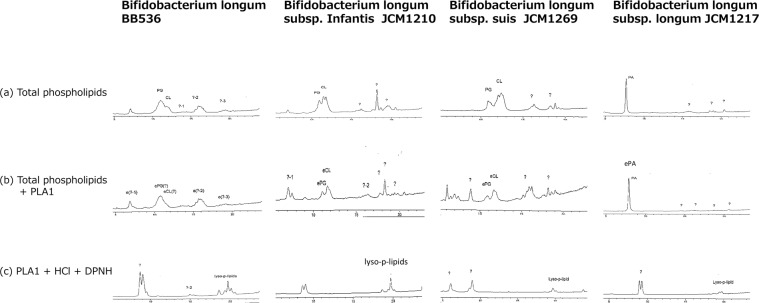
Chromatograms of phospholipids from *B. longum species*. Most of the phospholipid peaks of the *B. longum* species remained after treatment with PLA1 (**b**), indicating that these phospholipids were ether phospholipids (**b**), and peaks of ether phospholipids disappeared after treatment with DNPH-HCl, indicating that these ether phospholipids were plasmalogens (**c**). Presence of lysophospholipid (lyso-p-lipid) on the chromatogram after HCl treatment also indicated presence of plasmalogens.?; unidetified lipids.

Chromatograms of *B. longum* BB536 were showed in Fig. 2. Phosphatidylglycerol (PG) and cardiolipin (CL) were the most abundant phospholipids, but phospholipid classes of the other peaks were not identified. Phospholipid chromatograms of *B. longum subsp. infantis* and *B. longum subsp. suis* were similar to those of *B. longum BB536* (Fig. 2). However, phospholipid chromatograms of *B. longum subsp. longum*were different from those of the other *B. longum* species (Fig. 2). The main phospholipid of *B. longum subsp. longum* was not PG nor CL, but it was phosphatidic acid (PA). The PA peak remained after hydrolysis with PLA1, indicating the PA peak was an ether phospholipid. Hydrolysis of total phospholipids with phospholipase A1 (PLA1) resulted in most of the phospholipids of these *B. longum* species including *B. longum subsp. longum* that remained on chromatograms (Fig. 2), which indicated that most of these main phospholipids of *B. longum* species were ether phospholipids. Treatment of the PLA1 hydrolyzed phospholipids with 2,4-dinitrophenylhydrazine-hydrochloride (DNPH-HCl) caused disappearance of main peaks on the chromatograms (Fig. 2), indicating that most of phospholipids of these *B. longum* species were plasmalogens. Appearance of lysophospholipid peaks (lyzo-p-lipids) after DNPH-HCl treatment (Fig. 2) also indicates presence of plasmalogens in these *B. longum* species.

### Absence of plasmalogens in *B. animalis*

**Figure 3:**
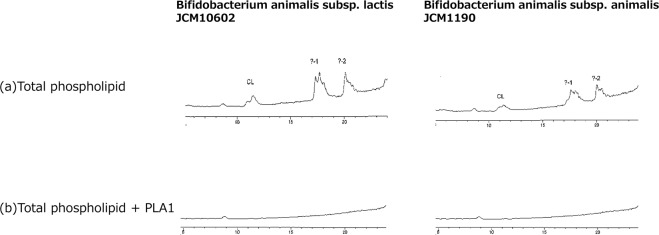
Chromatograms of *B. animalis* species. Chromatograms of phospholipids of the two *B. animalis* species were similar to each other (**a**), All of the chromatographic peaks disappeared after PLA1 treatment (**b**), indicating that all phospholipids of these *B. animalis* species were diacyl phospholipids.

Figure 3 shows chromatograms of phospholipids of *B. animalis subsp. lactis* JCM 10602 and *B. animalis subsp. animalis* JCM 1190. Chromatograms of these two species of *B. animalis* were identical (Fig. 3) and peaks of PG and CL were relatively small as compared to unknown peaks of the later retention times. Hydrolysis of these phospholipids with PLA1 caused disappearance of all peaks (Fig. 3), which indicated that all phospholipids peaks of these *B. animalis* species were diacyl phospholipids.

### Presence of plasmalogens in *Clostridium beijerinckii*

**Figure 4:**
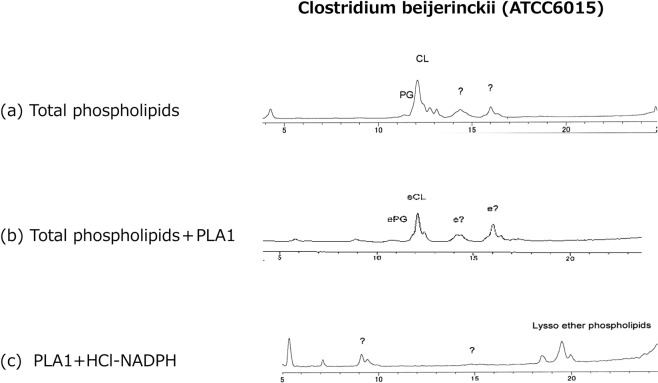
Chromatograms of phospholipids of *Clostridium beijinrickii*. Most of the phospholipids (**a**) remained after hydrolysis with PLA1 (**b**), indicating that these phospholipids were ether phospholipids. Treatment of these PLA1 treated phospholipids with DNPH-HCl resulted in disappearance of all major peaks and newly appearance of lyso-phospholipids (Lyso-p-lipids) (**c**).

Presence of plasmalogens in this *Clostridium* was already reported by the different methodology^15^. By the present method, a small peak of PG and large peaks of CL, and two large peaks of unidentified phospholipids peaks were observed (Fig. 4). Treatment of total phospholipids by PLA1 showed that a large part of CL peak and the other peaks including PG remained after PLA1 hydrolysis, indicating that these peaks were ether phospholipids. The peak of CL was somewhat changed by treatment with PLA1 which may indicate presence of diacyl type CL. Furthermore, the peaks after PLA1 treatments disappeared by DNPH-HCl, indicating that these ether phospholipids were plasmalogens. Appearance of lysophospholipids after HCl treatment also indicates presence of plasmalogens (Fig. 4).

### Occurrence of aldehydes from plasmalogens after acid hydrolysis

**Figure 5:**
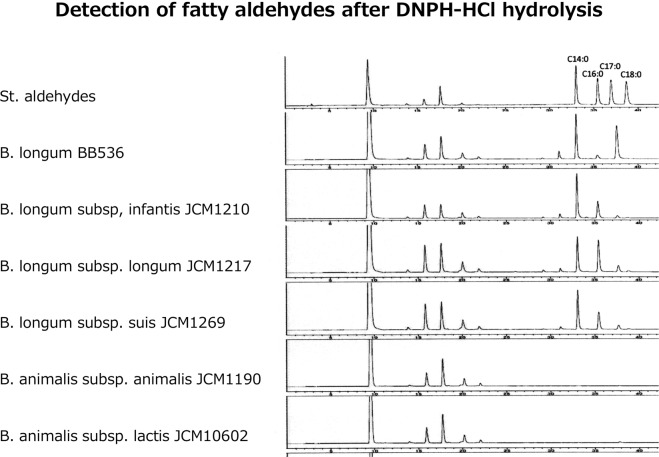
HPLC chromatograms of aldehydes from the indicated bacteria in which PLA1 hydrolyzed phospholipids were treated with DNPH-HCl. Peaks at early retention time were DPNH and non-specific short chain aldehydes. Chromatogram of standard long chain aldehydes was showed at the first chromatogram. Only *B. longum* species showed occurrence of aldehydes after acid treatment.

Occurrence of aldehydes after DPNH-HCl hydrolysis also indicates presence of plasmalogens in *B. longum* species. Figure 5 shows HPLC chromatograms of aldehydes after DNPH-HCl hydrolysis of PLA1 treated phospholipids of different bacteria. Peaks of short retention time were nonspecific short chain aldehydes. Peaks of the later retention times, which were seen in *B. longum BB536, B. longum subsp. infantis and B. longum subsp. longum, and B. longum subsp. suis* indicate aldehydes generated from plasmalogens, but *B. animalis* species showed no occurrence of aldehyde after hydrolysis with DNPH-HCl treatment of total phospholipids. Among the aldehydes generated from plasmalogens of *B. longum* species, C:14 aldehyde was the highest in all species of *B. longum*, and a small amount of C:12 and C:17 was found in all species of *B. longum* examined.

### Fatty acid composition of total lipids from *B. longum* BB536

Fatty acid composition of *B. longum* BB536 and *B. anilalis* subsp animalis JCM 1190 was showed in Table 1. By the method used in the present study, an odd numbered fatty acid (C17:0) was observed in both *B. longum* and *B. animalis* species. No polyunsaturated acids were detected.

**Table 1:** Fatty acid composition of total lipid extracts from *B. longum BB536* and a *B. longum animalis* species.

Fatty acid composition of total lipids (%)		
	*B. longum* BB536	*B. animalis* sucsp
		animalis JCM 1190
C12:0	1.2	1.1
C14:0	15.4	6.2
C16:1	4.5	4.5
C18:2		
C16:0	52.4	45.1
C18;1	2.1	13.2
C18:0	7.3	18.4
C17:0	13.3	8.1
Other Faty acid	3.9	3.5

Fatty aldehydes at the sn-1 position and fatty acids at the sn-2 position of phospholipids were included. No polyunsaturated fatty acids were observed,

### Presence of cardiolipin plasmalogen in *B. longum BB536*

**Figure 6:**
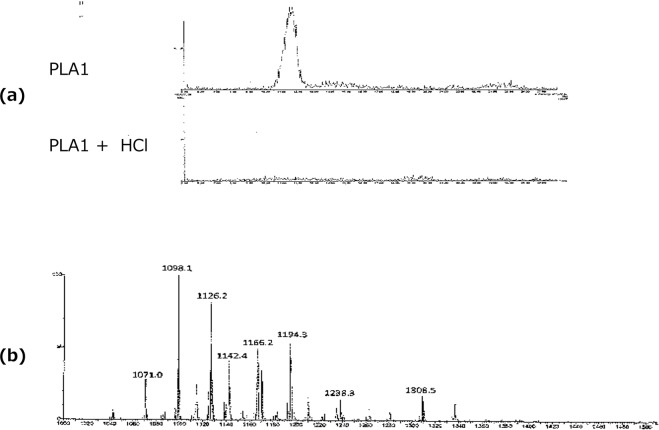
HPLC/MS/MS chromatograms of cardiolipin (CL) plasmalogen of *B. longum BB536*. The peak of cardiolipin ether lipid disappeared after HCl treatment (**a**), indicating that the peak was plasmalogens. MS spectrum of cardiolipin plasmalogen of *B. longum BB536* (**b**). PLA1; after PLA1 treatment, PLA1 + HCl; HCl hydrolysis after PLA1 treatment.

Presence of cardiolipin plasmalogen in *B. longum BB536* was confirmed by LC-MS/MS (Fig. 6a), and the peak disappeared after HCl treatment (Fig. 6a), indicating that the peak was plasmalogens. MS spectrum of cardiolipin plasmalogen of *B. longum BB536* was showed in Fig. 6b.

### Presence of phosphatidic acid plasmalogen in *B. longum subsp. Longum*

**Figure 7:**
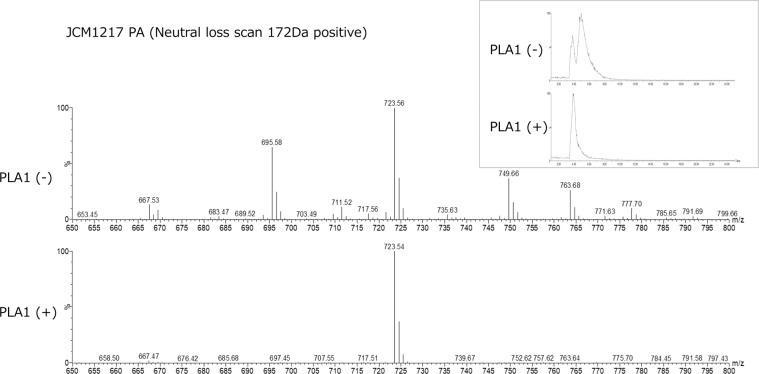
HPLC/MS/MS chromatogram of phosphatidic acid plasmalogens from *B. longum subsp. longum* JCM1217. Two peaks of total lipid extract of PA were reduced to one peak after PLA1 hydrolysis, indicating that the remaining peak was ether phospholipid (Insert). MS spectrum of this peak indicated that PA plasmalogen of this bacterium was 723, 54 Da. PLA1(+); after hydrolysis with PLA1.

Presence of phosphatidic acid (PA) plasmalogen in *B. longum subsp. longum* (JCM1217) was confirmed by LC-MS/MS (Fig. 7). One PA peak of HPLC-ELSD (Fig. 2) was divided into two peaks by scanning by LC/MS (Fig. 7) and one of the two peaks disappeared after PLA1 hydrolysis (Fig. 7), and the remaining peak was indicated to be PA plasmalogen (Fig. 7).

## Discussion

The present study showed presence of plasmalogens in some species of *B. longum* strain. Identification of plasmalogens in a *Clostridium* species with the same method may verify that the present methodology can identify plasmalogens.

After confirmation of the presence of plasmalogens in some *B. longum* species, we found a literature that indicated presence of plasmalogens in *B. animalis subsp. lactis*^23^. The report used gas chromatography-mass spectrometry (GC/MS) for detection of aldehydes after acid hydrolysis of total lipid extract from the bacteria. Aldehydes were observed after acid hydrolysis, but phospholipid class of the plasmalogen was not been identified^23^. However, we did not find any plasmalogens in *B. animalis subsp. lactis* (Fig. 3) nor generation of aldehydes after HCl hydrolysis of the phospholipids from the bacteria (Fig. 5).

Probably, both *B. longum* strain and *B. animalis* strain are the most important probiotic gut-microbiota in human^19–21^. Many beneficial effects of *Bifidobacterium* to human health have been reported^19–21,24–26^. It is interesting to note that plasmalogens were found only in *B. longum* species, but not in *B. animalis* species. It is not clear that there are any differences of probiotic effects to human health between *B. longum* and *B. animalis*.

Biosynthesis pathway of plasmalogens in anaerobic bacteria is different from that in animals^11–14^. In the animal tissues, biosynthesis of plasmalogen begins with acyl-CoA-dependent acylation of dihydroxyacetone-phosphate (DHAP) to form 1-0-acyl DHAP by DHAP acyl transferase which is a peroxisomal enzyme^13^. The acyl chain is then displaced by a long-chain fatty alcohol. In anaerobic bacteria, the dihydroxyacetone phosphate (DHAP) could not be an intermediate in the plasmalogen synthesis. Diacyl-glyceropho spholipids, phosphatidic acid (PA), cytidine5′-diphosphate (CDP)-diacylglycerol (DAD) and diacyl phosphatidyl glycerol (PG) may be precursors of plasmalogens in anaerobic bacteria^11,13,14^. The results of *B. longum* species in the present s tudy show the presence of PA, PG and Cl plasmalogens in this anaerobic bacteria.

Long-chain alcohols are utilized for biosynthesis of plasmalogens in animal cells; however, long-chain alcohols were not readily incorporated into plasmalogens in bacteria^11,14^. In contrast, long-chain aldehydes and fatty acids were readily incorporated into both the ether linked chains and acyl chains of plasmalogens in *Clostridium beijerinckii*^11,14^. Aldehydes generated from *B. longum* species included C:14, C:12 and C:17 aldehydes (Fig. 5). These aldehydes indicate aliphatic chains of the sn-1 position of plasmalogens in the *B. longum* species. Fatty acids analysis of total lipids from *B. longum* BB536 indicated that no polyunsaturated fatty acids and fatty alcohols were present in *B. longum* species (Table 1). On the other hand, C14:0 and C17:0 alkyl glycerols have been successfully used as alternative plasmalogen precursors^27,28^.

Many probiotic effects of *B. longum* to human health have been reported^20,29–33^. However, it is not known that these probiotic effects of *B. longum* are related to the presence of plasmalogens. Microbiota may control gut-brain axis^34–36^. Gut-brain axis is a bidirectional communication system between the gastrointestinal tract and the central nervous system^34–36^. Routes of this communication may include neural, immune, hormonal and metabolic pathway. The gut-brain axis may involve cognition, personality, mood, sleep, and eating behavior^34–36^. The gut microbiota plays a role in basic neurodegenerative processes such as the formation of the blood-brain barrier, myelination, neurogenesis, and microglia maturation, and it modulates many aspects of animal behavior^35–39^. Microbiota-gut-brain axis may relate to depression^38^. There have been many reports suggesting relationship between gut microbiota-gut-brain axis and Alzheimer’s disease (AD)^34–43^. There are also many reports suggesting abnormal relationship of gut-brain axis to Parkinson’s disease (PD)^44–49^.

Increased *Bacteroides* and decreased *Bifidobacterium* were observed in fecal samples of AD as compared to those of age- and sex-matched controls^39^. Gut microbiot a influences both the production and absorption of neurotransmitters such a s serotonin and GABA by increasing their bioavailability to the brain. Some components of the microbiota synthesize and release amyloid peptides and lipopolysaccharides, which in turn activate inflammatory signaling through the release of cytoki nes, with potential effects on pathophysiological cascade of AD^41^. The increased permeability of the gut and blood brain barrier induced by microbiota dysbiosis may mediate or affect pathogenesis of A D and other neurodegenerative disorders^42^. In addition, the microbiota can secrete large amounts of amyloids and lipopolysaccharides, which might contribute to the pathogenesis of AD^42,43^. Nutrients have been shown to affect the composition of gut microbiota as well as the formation and aggregation of cerebral amyloid-β^42^. Oral administration of *Bifidobacterium breve* strain A1 to AD mice reversed the impairment of attention behavior in Y maze test and the reduced latency time in a passive avoidance test, indicating prevention of cognitive dysfunction^43^.

Colonic bacterial composition is changed in PD^42^. Disturbance of a microbiota-gut-brain axis has been linked to specific microbial products that are related to gut inflammation and neuro-inflammation^44–46^. Fecal short chain fatty acid concentrations were significantly reduced in PD and short chain fatty acids from some gut-microbiota significantly reduced bowel symptoms in PD^47^. Gut-microbiota regulates motor deficits and neuro-inflammation in model of PD, suggesting that alterations in human microbiome represent a risk factor for PD^48^. Dysregulation of the brain-gut-microbiota axis in PD may be associated with the pathogenesis of PD itself^48^. Excessive stimulation of the innate immune system resulting from gut dysbiosis and/or small intestinal bacterial overgrowth and increased intestinal permeability may induce systemic inflammation, while activation enteric neurons and enteric glial cells may contribute to the initiation of alpha-synuclein misfolding in PD^49^.

All of the mentioned reports indicated a close relationship of microbiota-gut-brain axis to AD and PD. On the other hand, a close relationship between AD and plasmalogens has been indicated by the observations of decreased ethanolamine plasmalogen (plsPE) in the affected brain regions of AD^50–53^. Furthermore, decreased levels of plsPE in plasma or serum have been reported in AD^54,55^. Decrease of erythrocyte plasmalogen in AD was also indicated^56^. Decreased levels of plasma plasmalogens in PD was reported^57^. A plasmalogen precursor analog treatment reduced levodopa–induced dyskinesia in PD monkey^58^.

It has been indicated that chronic inflammation is involved in causes of AD and PD^3,5,6^. It is also reported that plasmalogens inhibit amyloid precursor protein procession by inhibiting γ-secretase activity^59,60^. We reported that oral ingestion of plasmalogens showed anti-inflammatory/anti-amyloidogenic effects of plasmalogens in lipopolysaccharide-induced neuroinflammation in adult mice^61^. These results were confirmed recently by the observation that oral ingestion of plasmalogens could attenuate the lipopolysaccharide-induced memory loss and microglial activation^62^. We previously reported that oral administration of purified ether phospholipids extracted from scallop improved cognitive functions of patients with AD^63^ and mild cognitive impairment (MCI)^64^.

Thus, many aspects of the reported functions of microbiota-gut-brain axis on AD and those functions of plasmalogens on AD are overlapped. Major phospholipid classes of plasmalogens in human tissues including brain are plsPE and plsPC, but major phospholipid classes of plasmalogens in gut microbiota are plsPG and plsCL. It is not known yet whether the presence of plasmalogens in *B. longum* strain is related to beneficial functions of *B. longum* strain on gut-brain axis to human health and human neurodegenerative diseases. The presence of plasmalogens in *B. longum* strain may play roles for many probiotic effects of *B. longum* to human health.

## Materials and Methods

### Methodology for identification of plasmalogens

Phospholipase A_1_ (PLA1) hydrolyzes acyl bond of the sn-1 position of glycerophospholipids, but it does not act on alkenyl and alkyl bond of phospholipids. Therefore, treatment of total lipids from bacteria with PLA1 leaves only intact ether phospholipids of all classes of glycerophospholipids. On the other hand, only alkenyl bond (vinyl ether bond) is susceptible to acid hydrolysis among the three subclasses of glycerophospholipids. Therefore, it is possible to identify glycerophospholipid subclasses including alkylacyl and alkenylacyl phospholipids by combination of PLA1 hydrolysis and acid hydrolysis of glycerophospholipids. Phospholipid classes were separated by HPLC-ELSD method which was developed previously by us^65,66^. Lysophospholipids generated by acid hydrolysis of plasmalogens could be detected by the HPLC-ELSD method, and aldehydes generated from plasmalogens by acid hydrolysis were labelled with DNPH and were detected by another HPLC method with UV detector.

### Materials

Phospholipase A_1_ (Phosphatidate 1-acylhydrolase, E.C. 3.1.1.32) from *Aspergillus oryzae* was purchased from Mitsubishi Kagaku and Foods Co. (Tokyo, Japan). The enzyme preparation consisted of 25% phospholipase A_1_ and 75% of dextrin, and its enzymatic activity was reported to be 10,000~13,000 units/g. Authentic phospholipids were purchased as a phospholipid kit from Sigma-Aldlich Co. through Funakoshi Co. (Tokyo, Japan); phosphatidylethanolmine (PE) from egg yolk, phosphatidylcholine (PC) from bovine liver, phosphatidylserine (PS) from porcine brain, lysophosphatidylethanolamine (LPE) from egg yolk, lysophosphatidylcholine (LPC) from bovine liver, cardiolipin (CL) from bovine heart, phospsphatidyl glycerol (PG) was synthetic substance (Phosphatidyl Dioleoyl), cerebrosides (total two spots) from bovine liver. Authentic long chain aldehydes were obtained from Tokyo Kasei Co. (Tokyo, Japan). All solvents used for HPLC analysis were HPLC grade and were obtained from Wako Pure Chemical Industries, Ltd. (Osaka, Japan). DNPH-HCl solution was purchased from Sigma-Aldrich Co.

### Cultivations of bacteria

Some of anaerobic bacteria in several different Yogurts and in powdery supplements, which were commercially available in market places, were cultured in TOS Propionate Agar Medium (Yakult Co. Japan) under anaerobic condition using AnaeroPak-Anaero (Mitsubishi Gas Chemical Co, Japan) at 40 °C for 2 days. Several single colonies were isolated and cultured again under the same condition. The Isolate was identified using amplification and subsequently sequencing of 16S rRNA gene using oligonucleotide primers 518F and 800R. One of the *Bifidobacterium longu*m was *Bifidobacterium* BB536, which was cultured from yogurt made by Morinaga Milk Co (Japan). *Bifidobacterium longum subsp. longum* JCM1217, *Bifidobacterium longum subsp. Infantis* JCM1210, *Bifidobacterium longum subsp. suis* JCM1269. *Bifidobacterium animalis subsp. animalis* JCM1190 and *Bifidobacterium animalis subsp. lactis* JCM10602 were provided by RIKEN BRC through the National Bio-Resource Project of the MEXT/AMED, Japan. These bacteria were cultured in TOS Propionate Agar Medium (Yakult Co. Japan) under anaerobic condition using AnaeroPack-Anaero (Mitsubishi Gas Chemical Co. Japan) at 40 °C for 2 days. *Clostridium beijerinckii* (ATCC8015) was purchased from American Type Culture Collection (ATCC), and was cultured by reinforced clostridium medium (Oxoid CMO149) with 1.5% agar under anaerobic conditions using AnaeroPak-Anaero at 37 °C for 2 days.

### Lipid extraction from bacteria and detection of phospholipids

Colonies of bacteria on culture plate were collected into a glass tube by scraping off, and bacteria were collected by centrifugation. Total lipids of bacteria were extracted by the method of Bligh & Dyer^67^. Briefly, 1 mL of water was added to the pellet and sonicated for 30 sec, and 3.75 mL of chloroform/methanol (1:2, v/v) were added, after vigorous mixing, the tubes were placed at room temperature for 30 min. 1.25 mL of chloroform was added, then 1.25 mL of water added. After brief centrifugation, chloroform layer was transferred to a glass tube and remaining aqueous layer was re-extracted with 1 mL chloroform. Combined chloroform layer was evaporated with N_2_ gas and re-suspended in hexane/isopropanol (3:2, v/v) before injection into the HPLC system^65^.

### Hydrolysis of total lipid with PLA1

An aliquot of total lipids was hydrolyzed with PLA1^65^. Hundred (100) mg/mL of the enzyme preparation containing 25% PLA1 was prepared with 0.1M citrate buffer (pH 4.5), and 1 mL of the enzyme solution was added to the total lipid extract. After being emulsified by an ultrasonic bath, the suspension was incubated at 45 °C for 60 min. The lipids were extracted with chloroform-methanol method described above^67^ and combined chloroform layer was dried under N_2_ gas. The lipids were reconstituted with hexane/isopropanol (3:2, v/v) before injection into the HPLC-ELSD system^66^.

### Acidic hydrolysis of plasmalogens

Acidic hydrolysis of alkenylacyl phospholipids (plasmalogens) was done by DNPH-HCl solution. Briefly, 0.5 mL of the DNPH-HCl solution was added to the dried ether phospholipids after PLA1 hydrolysis. After 20 min at room temperature, the hydrolyzed phospholipids were extracted by the method described above^63^ and the chloroform layer was dried under N_2_ gas. The dried lipids were reconstituted with hexane/isopropanol (3:2 v/v) and an aliquot was injected into HPLC^65,66^.

### Detection of aldehydes generated from plasmalogens by HPLC

HPLC system was an Agilent 1200 series, which consisted of quaternary pump, autosampler, and diode array detector. Detection of DNPH-aldehyde was done by 356 nm UV. Column was an XBridge BEA C18 (3.0 × 150, 2.5 µm, Waters Co). Flow rate was 0.3 mL/min and column temperature was 35 °C. Mobile phase A was acetonitrile and mobile phase B was 5 mM ammonium acetate. Gradient elution was done, 0–5 min 90% A, 5–20 min 100% A. 100% A decreased linearly to 90% A in 5 min and maintained 90% A until 30 min. The turnaround time was 30 min. An aliquot of the lipids after HCl-DPNH hydrolysis was injected.

### Analysis of fatty acid composition of total lipids from *B. longum* BB536

Total lipids from *B. longum BB536* were saponified with 0.5MKOH in methanol at 70 °C for 60 min. and fatty acids were extracted with hexane. The fatty acids were labelled with 9-anthrildiazomethane (ADAM). ADAM was prepared according to the method reported by Yoshida *et al*.^68^. The fatty acids were analyzed by using a column of Supersphere 100 RP-18 (250 × 4 mm, 4 *μ*m, Merck) with mobile phase A; acetonitrile, B; ethanol, C; hexane. A linear gradient of acetonitrile to A/B/C (30:40:30) was generated over 20 min. The flow rate was 1 mL/min and column temperature 30 °C. Peaks were detected at the excitation and emission at 365 and 412 nm, respectively^69^.

### Identification of PA, PG and CL by LC-MS/MS

LC-MS/MS system was a Waters system consisted of Acquity UPLC H-Class and Xevo TQ-S micro. Column used was KINNETEX HILIC (100 × 4.6 mm, 2.6 µm, Waters Co.). Mobile phase A;10 mM ammonium formate in water containing 0.5% formic acid, and mobile phase B; isopropanol/acetonitrile (5:2) containing 10 mM ammonium formate and 0.5% formic acid. Gradient elution; 5% A was increased to 50% in 20 min, 50% A was kept until 23 min and decreased to the initial condition in 2 min. Flow rate was 0.3 mL/min and column temperature was 50 °C. PA and PG was detected by neutral loss scan at 172 Da in positive mode. CL was detected by precursor ion scan at 153 Da in negative mode.
